# Systematic review of independent pharmacist prescribing for the treatment of cardiometabolic factors in chronic non-communicable diseases

**DOI:** 10.1136/ejhpharm-2025-004554

**Published:** 2026-02-26

**Authors:** Kevin Vowles, Christopher Garland, Maura McCarron, Michael G Scott, Cathy Harrison

**Affiliations:** 1 School of Psychology, Queen’s University Belfast, Belfast, UK; 2 Northern Ireland Department of Health, Belfast, UK; 3 Pharmacy, Medications Optimisation Innovation Centre, Belfast, UK

**Keywords:** PHARMACISTS, EVIDENCE-BASED MEDICINE, PHARMACY SERVICE, HOSPITAL, PRIMARY HEALTH CARE, PUBLIC HEALTH

## Abstract

**Objectives:**

Changes in health regulations in many developed countries have expanded prescribing privileges from medical doctors to other health professionals, notably nurses and pharmacists working in hospital and general practice settings. While some of these prescribers operate in a supervised capacity, increasingly there are those who operate as autonomous prescribers. This development will likely increase capacity for addressing common health challenges in the future. In the present systematic review we evaluated the evidence of independent pharmacist prescribing (IPP) in the treatment of metabolic targets associated with chronic non-communicable diseases (cNCDs), which are common, costly and often diminish quality of life.

**Methods:**

Published evidence evaluating the effects of IPP on cNCDs was reviewed. English language studies evaluating IPP for adults with these conditions and published since 2000 were included. Two reviewers independently evaluated papers for inclusion, IPP effectiveness and risk of bias.

**Results:**

A total of 11 studies were included, which examined IPP for three metabolic targets related to cNCDs—namely, blood glucose, blood pressure and cholesterol. Seven randomised controlled trials (RCTs) provided robust support for IPP in the treatment of hypertension compared with usual prescriber care. For diabetes there was modest support for glycaemic control including one RCT and two non-randomised evaluations. For hypercholesterolaemia, there were two RCTs, both of which were not specifically targeted at cholesterol reduction and yielded unclear results.

**Conclusion:**

Given that hypertension is one of the major risk factors for morbidity and mortality, expansion of care for this condition to include IPPs embedded in both hospital and community settings has marked potential for public health benefit. Although available evidence supports the feasibility of IPP for glycaemic control, more adequate evaluations are needed to determine safety and efficacy. Finally, IPP for hypercholesterolaemia likely requires additional investigation, including studies specifically targeting change in cholesterol.

**WHAT IS ALREADY KNOWN ON THIS TOPIC**

- Chronic non-communicable diseases are one of the leading causes of global morbidity and mortality. The effectiveness of independent pharmacist prescribing (IPP) in their treatment is not known.

**WHAT THIS STUDY ADDS**

- The evidence base for IPP is fairly robust for hypertension. There are initial indicators of effectiveness for diabetes and unclear results for hypercholesterolaemia.

**HOW THIS STUDY MIGHT AFFECT RESEARCH, PRACTICE OR POLICY**

- Pharmacists seem well poised to effectively treat hypertension, which has high potential for public health benefit given the broad range of health risks it poses. Further work is needed to clarify the results for diabetes and hypercholesterolaemia.

## Introduction

 Chronic non-communicable diseases (cNCDs) are common, costly, increase the risk of morbidity and mortality and are routinely associated with reductions in quality of life.^1–3^ The negative impact of cNCDs on quality of life globally has increased since the turn of the century, with a recent report from the World Health Organisation (WHO) noting that 100 million more healthy life-years were lost in 2019 compared with 2000.^4^ Their combined direct and indirect costs are predicted to be in excess of US$47 trillion per year by 2030.^5^ Given their high prevalence and substantial financial and social costs, adequate intervention for cNCDs is a top healthcare priority globally.

Some cNCDs can be effectively treated by pharmaceutical management of specific metabolic targets along with low intensity education or information provision. Specifically, the WHO identified three key metabolic issues that can be targeted with prescription medication, including hypertension, hyperglycaemia and hyperlipidaemia (including hypercholesterolaemia).^6^ Thus, the burden and cost of cNCDs could be substantially reduced if safe and appropriate access to these medications was more readily available. Historically, access has required a prescription from a medical doctor, although many countries have been broadening access through ‘non-medical prescribers’—typically nurses and pharmacists with specific training to enable safe and effective prescription independent of medical doctor supervision.

In addition to hospital settings, pharmacists are well suited to serve as non-medical prescribers in a community role, given that community pharmacies are often widespread and accessible in the developed countries of the world. In recognition of the potential for expanding the role of independent pharmacist prescribing (IPP), several countries have been active in developing practice models and training guidelines.^7 8^ For example, in 2006 the UK Department of Health published guidance on ‘non-medical prescribing’ which was primarily directed at nurses and pharmacists working within the National Health Service.^9^ Recent reforms to the initial education and training of pharmacists trained in the UK mean that all newly qualified pharmacists will register as independent prescribers from September 2026. This emerging prescribing-ready workforce offers the potential for more timely access to prescribed medicines for cNCDs, as prescribing pharmacists are already in place in some UK healthcare settings and will be more broadly in the workplace from 2026 onwards. In particular, community pharmacies may be an ideal facility for addressing at least some cNCD-related metabolic targets as they are accessible, prevalent and are already an established part of primary healthcare services.

Even with the significant potential inherent in expanding the treatment of cNCDs to prescribing pharmacists, there is an absence of published evidence regarding several aspects of IPP for metabolic targets relevant to cNCDs, including evaluations of effectiveness and suitable health conditions. Furthermore, given changes to non-medical prescribing legislation across various jurisdictions, an up-to-date review of evidence is needed to inform policy implementation and practice structure. Thus, we performed a systematic review to evaluate the effectiveness of IPP for cNCD metabolic targets, particularly in comparison to usual medical prescriber care.

## Methods

### Search strategy

First, a strategy was developed in relation to search terms and the identification of relevant databases. It was planned that initial testing of search terms would use the PubMed database to evaluate accuracy of identification. Search term development used the Population, Intervention, Comparison, Outcomes (PICO) framework as follows: pharmacists (population) independently prescribing medications (intervention) to treat the metabolic targets of cNCDs (outcome). We did not pre-specify a comparison within this framework given that our plan was to include both cohort and controlled studies. Search terms were evaluated for appropriateness and endorsed by a research librarian.

Specific search term development included four broad categories to include (1) independent practice; (2) pharmacist involvement; (3) medication prescribing; and (4) cNCDs. We elected to use broad search terms and additive Boolean operators within each of these four categories (ie, OR) and restrictive operators (ie, AND) across them to maximise identification of relevant literature. Further, it was planned that we would adopt a search strategy with broad inclusion terms based on the assumption that searches would yield a large number of articles requiring closer scrutiny to adequately evaluate inclusion and exclusion criteria.

The initial search took place on PubMed and Google Scholar (final search date 25 April 2024), with additional searches conducted on the National Library of Medicine’s MedlinePlus, the Cochrane Database of Systematic Reviews and reference list searches of articles to identify relevant papers. Prior to conducting the search we were aware of at least one meta-analysis on non-medical prescribing^10^ and planned to review all studies it included. Following a review of published articles, we also searched the ‘grey’ literature of conference abstracts and journal reports through Medline and International Pharmaceutical Abstracts.

### Inclusion criteria

#### Types of studies

Both cohort and randomised controlled trials (RCTs) were included. Articles had to be published in English and published in 2000 or after. This latter criterion was intended to capture data specifically related to the altered prescribing regulations allowing for IPP.^11 12^

#### Types of interventions and participants

Studies had to provide an evaluation of treatment outcomes in relation to IPP. The definition of IPP used was pharmacist-led determination of prescribed medication dosing for participants with target cNCD. Thus, studies that included only pharmacist-led education with medication feedback or medication recommendations made to another prescriber were excluded. The definition of cNCD included conditions that required long-term management and ongoing care from healthcare providers for a defined health condition. Finally, data were included regardless of practice setting (eg, inpatient, outpatient, community).

#### Selection of studies

Several sequential evaluation stages were planned to identify articles and extract information, including initial title review, abstract review and full text review. Two reviewers (KV and MM) independently evaluated articles at each stage. Disagreements in reviewer ratings were planned to be initially addressed via discussion to achieve consensus. When consensus could not be achieved, we planned to either retain the article for the next stage of review to allow greater scrutiny or to use additional independent reviewers from the author team to achieve consensus. In cases where criteria for inclusion could not be assessed adequately (eg, when the title or abstract did not provide sufficient information), articles were retained for subsequent stages of review until such time as criteria could be adequately evaluated. Information on type of cNCD, metabolic target, intervention/control condition details and outcomes for each study was extracted during full-text review separately by each reviewer.

### Analytical plan

As noted, the overall goal of this work was to evaluate the current state of knowledge in relation to IPP for cNCDs. Relevant guidelines such as Synthesis Without Meta-analysis (SWiM)^13^ were used to develop a four-step analytical plan, as detailed below.

First, we planned that selected studies would be evaluated based on individual cNCD groupings (eg, hypertension, diabetes) to allow a determination of overall effectiveness in relation to specific metabolic targets. We planned that a common metric for evaluation would be used for each cNCD based on the target of IPP treatment (eg, blood pressure for antihypertensives; blood glucose for medications aimed at glycaemic control).

Second, where possible we planned to prioritise evaluation and synthesis of quantitative outcome evidence. For example, we anticipated that quantitative data could include numerical evaluation of treatment effectiveness (eg, mean improvement in blood pressure or blood glucose) or the proportion of participants achieving treatment success in the form of pre-specified treatment targets (eg, blood pressure or blood glucose findings below identified risk thresholds).

Third, where quantitative synthesis was not possible due to lack of information or heterogeneity in variables reported across studies, we planned to use narrative synthesis^13^ to summarise outcomes of IPP within each cNCD. Specifically, narrative synthesis involves a description of methodological details and outcomes from each study, heterogeneity and homogeneity in approach across the study and summaries for each cNCD in relation to IPP.

Fourth, we evaluated methodological quality for randomised and controlled studies. Two reviewers (KV and MM) independently evaluated the risk of bias using the Cochrane Risk of Bias 2.0 (RoB2) tool.^14^ It was planned that any disagreements in assessment would initially be addressed through discussion and, if consensus could not be reached, a third reviewer would review the study details to serve as tie breaker. When articles did not provide sufficient assessment information to complete the RoB2 domains, the corresponding authors were contacted to clarify the study details. In total, additional details were requested and provided from corresponding authors for three studies.^15–17^

## Results

The final search terms are shown in box 1 and figure 1 shows the PRISMA diagram detailing the flow of information throughout the review stages. In total, 1199 articles were identified across all included databases, which was reduced to 765 articles after removing duplicates. Thus, initial title screening proceeded with these 765 articles. Figure 1 also shows the reasons for exclusion at the abstract and full-text review stages. The primary reasons for exclusion included the absence of data (eg, commentaries, study protocol descriptions), absence of metabolic target outcome analysis (eg, feasibility and acceptability intervention only) and absence of IPP (eg, prescription was supervised or collaborative; intervention was educational). In total, 24 articles were reviewed in full with 11 retained for review and data synthesis. Across all three stages of the review initial inter-rater reliability was excellent (title review kappa 0.80; abstract review kappa 0.84; full-text review kappa 1.0). Consensus was achieved following discussion for all articles that had initial disagreement in reviewer inclusion decisions.

Box 1 **Literature search strategy**

**Terms and Boolean operators**

((non-medical) OR (nonmedical) OR (independent)) AND

((pharmacist) OR (pharmacy)) AND

((prescribing) OR (prescriber) OR (prescription)) AND

((chronic) OR (persistent) OR (persisting) OR (non-communicable) OR (noncommunicable))

**Figure 1:**
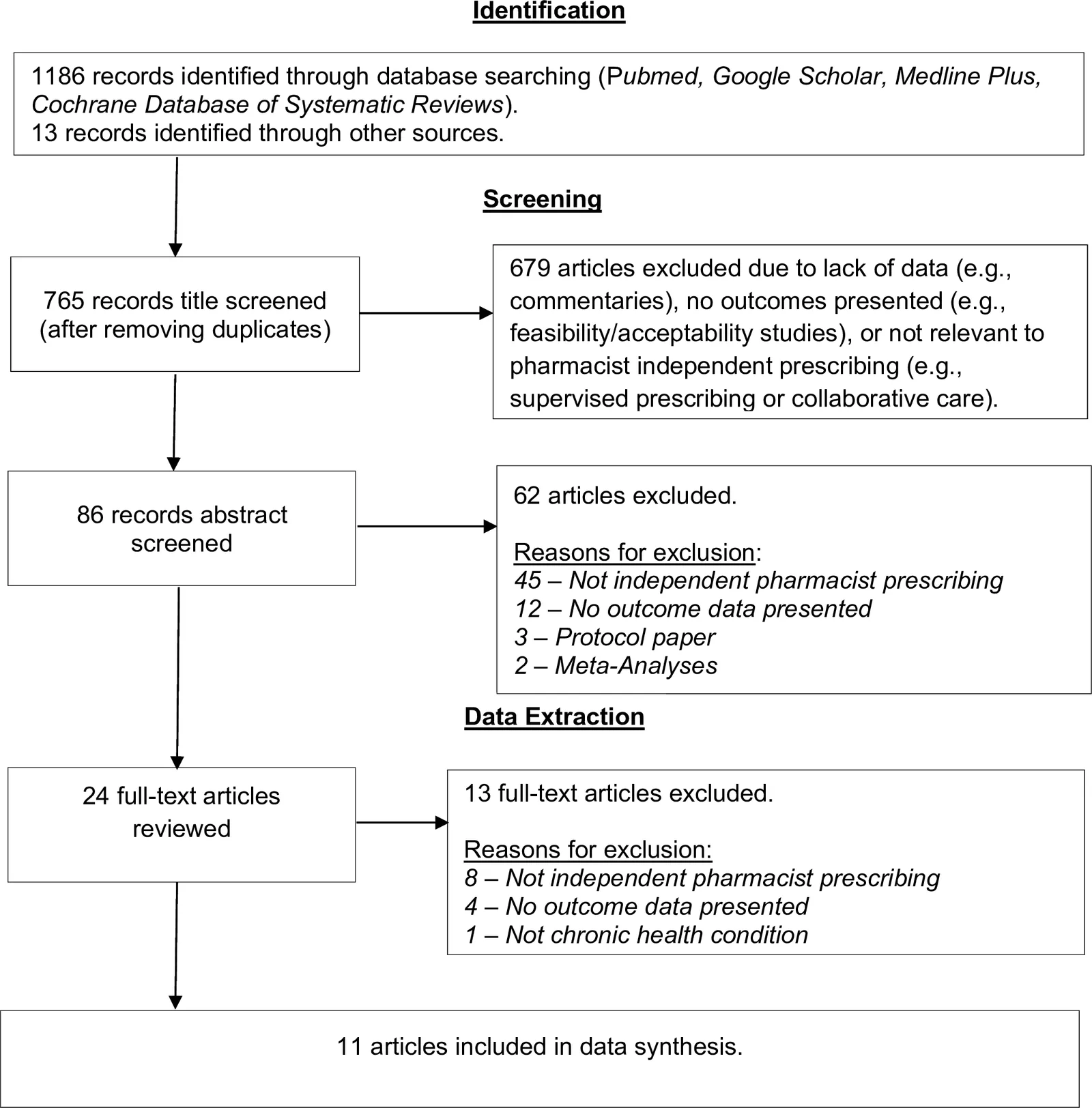
Flow of information through the different phases of the review, as specified by the PRISMA statement.

With regard to the grey literature, the search for relevant reports of IPP and of conference abstracts with unpublished information did not yield any additional studies. A search of the International Pharmaceutical Abstracts database did not identify any abstracts with the search terms used in the scientific paper review. The review of reports and policy papers yielded 121 results; however, none of them met the inclusion criteria as no new data were presented in any of these documents.

### Initial assessment of chronic health condition grouping

Across the 11 articles included in the review, IPP for three metabolic targets related to cNCDs was evaluated, which included blood pressure (BP), blood glucose and cholesterol. Although some studies included more than one target (eg, BP and glucose), for the purpose of this review each target was reviewed separately.

Following the review of all articles, quantitative synthesis was only possible for the trials of BP as heterogeneity in methods, approach and outcomes differed significantly for blood glucose and cholesterol, which allowed narrative synthesis only. Furthermore, only the studies targeting BP included RCTs to allow for bias analysis using RoB2. The remaining studies were an uncontrolled cohort study,^18^ a 78-week follow-up of the uncontrolled cohort study,^19^ a retrospective chart review^20^ and a secondary analysis of cost-effectiveness.^21^

### Blood pressure

In total, seven of the 11 trials examined the efficacy of IPP of antihypertensives in comparison to usual care.^15–17 22–25^ An additional paper^21^ provided a secondary cost-effectiveness analysis of one of these studies.^25^

Five of these trials^16 17 23–25^ had a large overlap in terms of methods, with all of them targeting individuals with high BP for inclusion. All included a comparator arm of usual primary care provider prescribing and most supplanted usual care prescribing with provision of written BP educational materials or single episodes of pharmacist consultation without follow-up. In the intervention arm, all studies included more than IPP alone. At a minimum, the IPP group included 30 min meetings between pharmacist and participant every 3 months for a year plus follow-up meetings as required,^16^ while other trials included remote BP monitoring, monthly meetings and/or an intervention taper once BP targets were met.

All five studies provided the percentage of participants who achieved target BP levels of <130/80 mmHg for those with diabetes and/or chronic kidney disease and <140/90 mmHg for all other participants. The consistency across studies therefore allowed for a summary of quantitative information which is shown in table 1. As shown in the table, across follow-up periods ranging from post-treatment through 12 months following study inception, receipt of IPP was associated with a larger proportion of participants achieving target BP (range 54–81%) compared with usual care alone (range 30–53%).

**Table 1:** Percentage of participants who achieved blood pressure targets* at follow-up for independent pharmacist prescribing (IPP) and usual care

Study	Assessment timing	IPP	Usual care
Cohen *et al*,^15^ 2011	6 months	58%	33%
Hirsch *et al*^16^ 2014	6 months	81%	44%
Magid *et al*,^23^ 2013	6 months	54%	35%
Tsuyuki *et al*,^25^ 2015	6 months	58%	37%
Margolis *et al*,^24^ 2013	6 months	72%	45%
	12 months	71%	53%
	Both 6 and 12 months	57%	30%
Vivian,^17^ 2002	Post-treatment	81%	30%

* Cohen *et al*: systolic blood pressure <130 mmHg. All other studies: <140/90 mmHg or <130/80 mmHg for those with diabetes/chronic kidney disease.

The study by Cohen and colleagues^15^ included US military veterans with type 2 diabetes. This study was also distinctive in that its treatment condition consisted of 8 hours of multidisciplinary education classes over 1 month followed by 5-monthly 90 min booster sessions with a pharmacist in comparison to usual primary care as the control. Outcome measures for the trial included all three metabolic targets included in this review (BP, blood glucose and cholesterol). With regard to BP, target blood pressure was <130 mmHg systolic BP (SBP), which was achieved by 58% of individuals in the intervention arm and 33% of individuals in the control arm, findings that are within the range of outcomes observed in the other trials of IPP for hypertension (table 1).

The trial by McAlister *et al* included participants with post-ischaemic stroke or transient ischaemic attack.^22^ Both trial arms included nurse-led case management including lifestyle advice, BP and cholesterol assessment, smoking cessation advice and medication adherence checking. The intervention arm also included IPP. The targeted outcomes of the trial included both a BP and cholesterol target, <140 mmHg SBP and low-density lipoprotein <2.0 mmol/L, with trial success reported as individuals who achieved both. Descriptively, 43.4% of those in the IPP group achieved both targets compared with 30.9% in the control group, a significant difference. The post-stroke nature of the sample is noteworthy, however, as almost two-thirds of participants were at the SBP target before the trial began. Thus, it is difficult to gauge the clinical significance of the IPP intervention in isolation.

Finally, a secondary cost-effectiveness analysis by Marra *et al* indicated good evidence of savings and clinically relevant benefit^21^ (primary study by Tsuyuki *et al*^25^). Using the average SBP reduction in the trial of 18.3 mmHg, the estimated impact was fewer cardiovascular events per person per year, which in turn supported outcomes of both longer life-years and more quality adjusted life-years. The lifetime savings were estimated to be $C6364 per individual, rising to more than $C11 000 per individual in models that assumed stable medication costs and provision of follow-up care.

#### Bias assessment

The RoB2 was used to assess the risk of bias in the seven hypertension RCTs.^15–17 22–25^ As shown in figure 2, risk of bias was deemed to be low across most domains for all seven RCTs, with some concerns noted only for a single domain in two studies.^24 25^ For both studies the outcome assessors were not blinded to participant treatment condition assignment. No other concerns were indicated across included studies.

**Figure 2:**
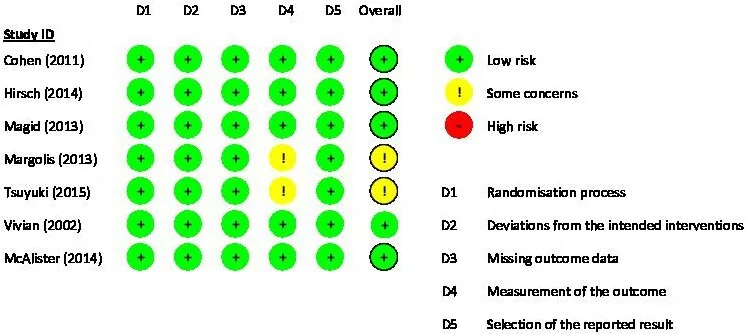
Risk of bias evaluation outcomes for each randomised controlled trial.

### Blood glucose

Three individual studies examined IPP for managing haemoglobin A1c (HbA1c) levels in people with diabetes,^15 18 20^ with one including long-term follow-up assessment in a separate report.^19^ The intervention content of the studies was variable, as was the inclusion of a usual care comparator group, so they are reported individually here and are summarised via a narrative synthesis.

The multifaceted study by Cohen and colleagues in US veterans with diabetes^15^ described above included IPP for blood glucose with an identified treatment target of <7% HbA1c (<53 mmol/mol). At the 6-month follow-up assessment a significantly greater proportion of individuals in the intervention group achieved this target in comparison with usual care control (41% and 20%, respectively).

The second study evaluating IPP for HbA1c was an uncontrolled cohort design including 100 individuals diagnosed with type 2 diabetes across 12 community pharmacies in Canada and included a post-treatment assessment only at 26 weeks post-baseline.^18^ The intervention was defined as a fixed titration of 10 units of insulin which participants were instructed to increase by one unit per day. This was provided in combination with education regarding insulin use and dose titration, along with blood glucose self-monitoring instructions. At 26 weeks post-baseline assessment there was a significant reduction in HbA1c (mean reduction 1.8% (95% CI 1.4% to 2.0%); 9.7 mmol/mol (95% CI 5.3 to 15.3)), with 51% of participants in the group having achieved the target HbA1c level of <7% (<53 mmol/mol). A subsequent follow-up study at 78 weeks post-baseline indicated a loss of treatment effect, which roughly equated to a loss of 50% of the HbA1c improvement, although the reduction remained statistically significant from baseline.^19^ The authors attributed the loss of treatment effect to the absence of structured pharmacist follow-up.

The third study by Bowron *et al* consisted of a retrospective review of IPP in comparison to medical doctor management among UK patients attending a hospital clinic for adult onset diabetes.^20^ The IPP was associated with no difference in HbA1c compared with medical doctor management. The trial report provided no details regarding intervention approach, specific values of HbA1c or patient variables, so it offered negligible utility in relation to the goals of this review.

Overall, while a small number of studies have examined IPP for diabetes, they are methodologically distinct from one another in terms of intervention specifics, degree of IPP and evaluation of outcomes. Thus, while there is support regarding the effectiveness of IPP, it is not possible to draw definitive conclusions.

### Hypercholesterolaemia

Two studies examined cholesterol following IPP. The multidisciplinary education and IPP trial by Cohen and colleagues^15^ found a non-significant between-group difference, although the α value approached the conventional cut-off point for significance (p=0.059). The target value for low density lipoprotein level was <100 mg/dL (2.59 mmol/L), which was achieved by 82% of the IPP intervention group and 65% of the control group.

The second trial was the post-stroke study by McAlister and colleagues.^22^ As noted above, while the IPP arm was associated with a greater proportion of individuals achieving both BP and cholesterol targets, combined outcomes were reported which rendered it impossible to separate the findings for each target individually.

As with the studies of IPP for glycaemic control in people with diabetes, the studies evaluated here substantially differed from one another in terms of target population, intervention details and design. Thus, there was little scope for narrative synthesis beyond concluding that there is a need for further work in this area.

## Discussion

With regard to the overarching aim of this systematic review, there were three specific cNCD metabolic targets for which there was at least some examination of IPP effectiveness. The evidence in support of IPP for hypertension was fairly robust in that all trials indicated good treatment effectiveness compared with usual care prescribing. In particular, five of the seven studies were similar in terms of methods and also had findings that were descriptively similar to one another.

For the other two targets the evidence was limited as the small number of published studies were heterogeneous in terms of methods, outcome measurement and level of detail provided in terms of study specifics. Specifically, there were only three trials examining IPP for glycaemic control in those with diabetes, and only one of these studies was controlled. While the results were promising as all three studies were supportive of IPP, further work is required before any definitive conclusions can be drawn.

No studies examined IPP for cholesterol as a primary outcome, as the two studies that evaluated change in cholesterol were for individuals with diabetes^15^ and who were post-stroke.^22^ The nature of the results of these studies also limits synthesis, as the difference between IPP and usual care was just above the conventional α level of p*<*0.05^15^ and was reported in a manner that mixed reporting of BP and cholesterol targets,^22^ so further work in this area is clearly necessary to determine IPP efficacy.

The IPP literature overall was fairly limited as only 11 papers were identified which examined efficacy in relation to cNCD metabolic targets and for only three metabolic targets. There was no other evidence found in relation to IPP for other cNCD metabolic targets. This result can be contrasted with the large body of literature supporting pharmacist involvement in a broad array of conditions in terms of the provision of counselling, medication review and education.^10 26 27^ Thus, it seems important to draw a distinction between pharmacist involvement in the treatment of cNCDs in terms of these latter areas of historic practice and direct provision of IPP. Given the changes in pharmacy practice in many countries internationally, the lack of evidence is noteworthy and can likely be considered as a clear priority for pharmacy researchers.

### Study limitations

There are several caveats to consider in relation to the data reviewed here and how they relate to IPP. First, while IPP is a fairly new phenomenon within the last 20 years, practice models are expected to change significantly in the next few years as the number of pharmacists with training in independent prescribing increases. If IPP is to be an evidence-based intervention, a substantial amount of work is necessary to examine the efficacy of IPP in practice and to optimise healthcare contribution, as well as to evaluate safety and identify limitations. There are case studies in making substantial changes to prescribing practice based on poor quality data, which indicate the significant risks involved. The ongoing ‘opioid crisis’ in many countries is a prime example, where poor quality data were used as a basis for profound changes in prescribing practices, which have contributed to a significantly increased risk of morbidity and mortality.^28 29^ Relatedly, there were no reports across any study reviewed regarding adverse events following IPP, and that is something that will be important to evaluate as this area progresses.

Second, the studies that met the criteria for inclusion were all published in 2015 or earlier. It is not clear why there have not been more recent studies in IPP and perhaps a new generation of evaluations would be useful in guiding both practice and policy. For example, the UK’s Royal Pharmaceutical Society^30^ designations for the increasing complexity of pharmacy practice from foundation to advanced levels do not appear to be an evidence-based distinction, and it may be wise to develop evidence as a guide in relation to the definition of that distinction.

Third, none of the studies involved consisted of prescription in isolation. Instead, prescription was accompanied by patient education, counselling and, in some cases, more intensive case management. Pharmacists may be ideally placed to provide this care, and it may be that these additive components of intervention are important predictors of effectiveness in pharmacy settings. A review of qualitative studies by Mills and colleagues on perceptions of IPP indicated that both foundational and advanced pharmacy prescribing require a greater commitment of time for assessment, treatment planning and ongoing care.^31^ For example, the assessment of symptoms and patient background details requires adequate contact time with patients, with time also required to document treatment plans and rationale.

Finally, there are other challenges related to IPP for pharmacists themselves, including concerns regarding prescribing scope, accountability, unclear role of diagnostic skills, and organisational and financial support, as well as the necessity for access to patient medical files.^32^ While examination of these latter issues falls outside the scope of the present review, they will likely impact the overall effectiveness, safety and acceptability of pharmacists prescribing medications independently.

## Conclusion

The overall evidence in the published scientific literature examining IPP for cNCD metabolic targets is fairly sparse at present and is somewhat disappointing given the substantial interest in this area. A noteworthy exception is for hypertension, where there is reasonable evidence to support the effectiveness and high potential for public health benefit.

## Data Availability

Data are available upon reasonable request.
